# Never Too “Old”: Insights From the Intergenerational Classroom

**DOI:** 10.1093/geroni/igaf122.2950

**Published:** 2025-12-31

**Authors:** Jessica Hsieh, Raza Mirza, Christopher Klinger, Alexis Hart, Florene Shuber

**Affiliations:** University of Toronto, Toronto, Ontario, Canada; University of Toronto, Toronto, Ontario, Canada; McMaster University, Hamilton, Ontario, Canada; University of Toronto, Toronto, Ontario, Canada; Christie Gardens, Toronto, Ontario, Canada

## Abstract

The topic of aging demands greater attention in education systems, and intergenerational approaches help to combat ageism and improve the lives of older adults, now and in the future. Taking an intergenerational approach to contextualizing the experiences of older adults is a step towards addressing challenges commonly associated with aging. The University of Toronto (UofT) partnered with Christie Gardens, a retirement community, to launch an innovative experiential learning initiative: The Intergenerational Classroom. Half the students were UofT undergraduates; the other half were older adults residing at Christie Gardens. Through interactive seminar-style discussions, collaborative projects and mentorship, the course, which was held at Christie Gardens, and offered in the Fall 2023 and Fall 2024 semesters, provided a semester-long exploration on aging. This study explored the impacts of the Intergenerational Classroom from the perspectives of undergraduate students and older adults who had participated in the program. Guided by a phenomenological qualitative methodology, this study conducted in-person, semi-structured interviews with a sample of undergraduate students (n = 10) and older adults (n = 16). To enhance trustworthiness, two researchers independently analyzed transcript data to identify key transcript statements into themes. Outcomes of program success were identified across domains related to lasting friendships and bonds, increased awareness of aging issues, reduced ageist attitudes, and community and civic engagement. The taxonomy developed provides a comprehensive and conceptually organized range of successful outcomes to serve as infrastructure for the development of meaningful intergenerational programming outcome measures.

